# Systemic sclerosis manifesting as intractable gastro-oesophageal reflux and diarrhoea: a case report from Kenya

**DOI:** 10.11604/pamj.2021.39.225.29771

**Published:** 2021-08-03

**Authors:** Gloria Wangechi Mugo, Eric Mwenda Murunga

**Affiliations:** 1 Gastro and Liver Centre, Nairobi, Kenya

**Keywords:** Systemic sclerosis, gastrointestinal system, Kenya, case report

## Abstract

Systemic sclerosis is a rare condition that has not been well reported in Africa, and several multisystemic manifestations, including gastrointestinal ones, have not been well documented locally. We present an unusual case of persistent gastro-oesophageal reflux and diarrhoea in a 74-year-old Kenyan female, who progressively developed abdominal distention, dysphagia and Raynaud´s phenomenon. Stool tests were unremarkable, whereas antinuclear antibody, ribonucleoproteins antibody (anti-nRNP/Sm) and anti-Sjögren's-syndrome-related antigen A autoantibody (anti-SSA) tests were positive. Endoscopic and imaging investigations revealed features of gastrointestinal dysmotility including reflux oesophagitis, gastroparesis and chronic intestinal pseudo-obstruction. A diagnosis of systemic sclerosis was made, and she responded well to medical treatment. We present this case to contribute to the limited literature of a disease associated with high morbidity and mortality, as well as encourage fellow clinicians to have a high level of suspicion in their differentials of persistent gastrointestinal dysmotility.

## Introduction

Systemic sclerosis is a rare autoimmune connective tissue disease characterized by abnormal excessive collagen deposition in the skin and internal organs, immune dysregulation and vasculopathy [1]. It typically affects young or middle-aged women and is classically associated with scleroderma [1]. Gastrointestinal sclerosis is the third most common manifestation of systemic sclerosis, and clinical evaluation, laboratory investigations and imaging are pertinent in establishing the diagnosis and detecting this visceral involvement [1]. This multidisciplinary condition can be effectively managed medically, thus preventing serious internal organ complications associated with high morbidity and mortality. We aim to increase awareness of the associated gastrointestinal dysmotility as this diagnosis can be overlooked or missed by clinicians.

## Patient and observation

**Patient information:** a 74-year-old female presented with persistent heartburn, acid reflux and up to 6 non-bloody loose stools per day. Her bowel movements were painless and unaffected by fasting or diet. She also reported bloating, flatulence, difficulty swallowing and unintentional weight loss of 17 kilograms. She was euglycemic, and her regular medication included telmisartan and amlodipine for blood pressure control.

**Clinical findings:** her vital signs were unremarkable, and she was cachexic with taut hyperpigmented skin on her face. She had a distended soft non-tender abdomen with no palpable masses.

**Figure 1:**
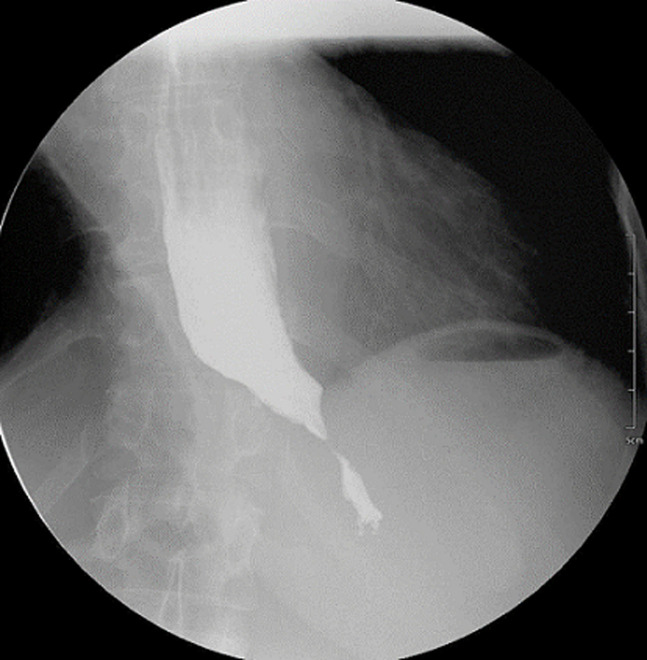
barium swallow study showing an abrupt tapering of the distal oesophagus at the gastro-oesophageal junction, with distal oesophageal dilatation; there was stagnation of the barium agent, indicating severe oesophageal dysmotility

**Figure 2:**
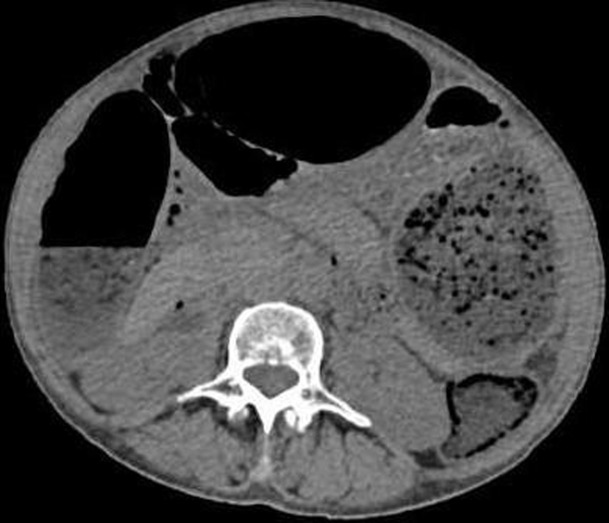
abdomino-pelvic CT scan in axial view, showing distension of the stomach and intestines; the distended large bowel anterior to the liver displaced it medially, suggestive of a long-standing process

**Figure 3:**
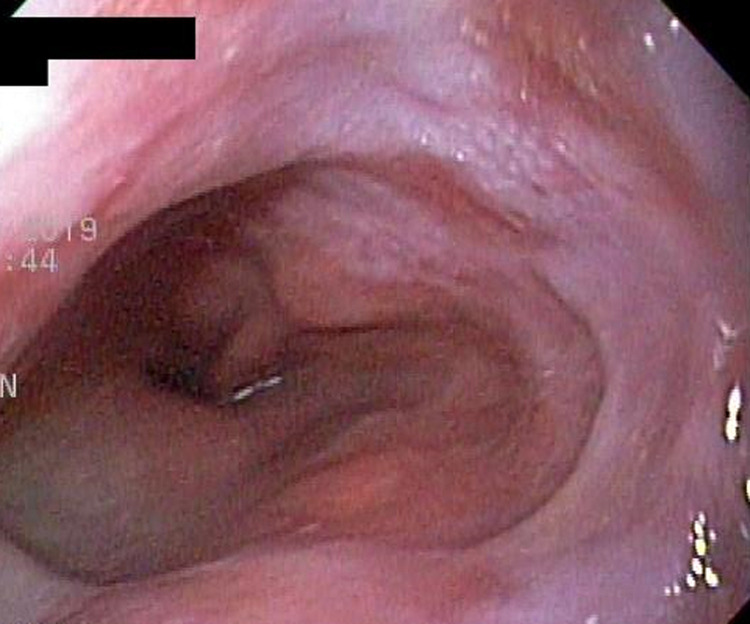
gastroscopy demonstrated a dilated proximal oesophagus with a distal narrowing and oesophagitis

**Figure 4:**
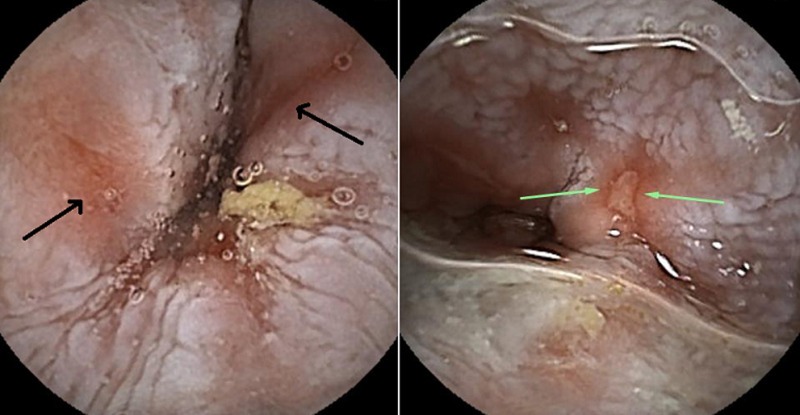
(A, B) capsule endoscopy images revealed distal oesophagitis with ulceration (green arrows)

**Diagnostic assessment:** laboratory tests showed mild microcytic anaemia and loose stools with pus cells. Stool studies for parasites, occult blood, elastase and faecal fat were all normal. Antinuclear antibody (ANA) was positive with a titre of 1: 320, and extractable nuclear antigen was positive for nRNP/Sm and SSA antibodies. Serum amylase, tissue transglutaminase antibody, anti-liver kidney microsomal antibody, anti-smooth muscle antibody and antimitochondrial antibody were normal. Barium swallow demonstrated proximal oesophageal dilatation with delayed emptying (Figure 1). A chest computed tomography (CT) scan showed the usual interstitial pneumonitis pattern of scleroderma-related lung disease. Abdominopelvic CT scans revealed luminal dilatation of the oesophagus, stomach, small and large bowel, as well as faecal loading (Figure 2). No cause of mechanical obstruction was identified. An upper endoscopy showed proximal oesophageal dilatation with distal oesophagitis and narrowing (Figure 3). Biopsies of the distal oesophagitis were mild non-specific chronic inflammation on histology, in keeping with gastro-oesophageal reflux disease (GORD), whereas duodenal biopsies revealed normal mucosa. There was no gross or histological evidence of malignancy, coeliac disease or telangiectasia. Capsule endoscopy revealed significant oesophageal dysmotility, with the capsule taking more than 5 hours to traverse the oesophagus despite administration of intravenous metoclopramide. Distal oesophagitis with ulceration was seen, and a significant amount of food was in the stomach despite overnight fasting, suggestive of gastroparesis (Figure 4(A, B)).

**Therapeutic intervention:** she was treated with antibiotics and rabeprazole/domperidone combination capsules for small intestinal bacterial overgrowth (SIBO) and dysmotility and reported marked improvement in her symptoms. She was then started on mycophenolate mofetil.

**Follow-up and outcomes:** the patient reported marked improvement in her symptoms with resolution of the loose stools, dysphagia and reflux, and she has monthly follow-ups in our clinic with monitoring of haemoglobin and inflammatory markers. Her long-term maintenance therapy includes regular antibiotic cycling for recurrent SIBO, mycophenolate mofetil, prednisone and esomeprazole/itopride slow-release capsules.

**Patient perspective:** “when I first came to Gastro and Liver Centre, I was very down. I was very uncomfortable and could not do simple things. It got so bad I had to stay home and have my family care for me, and although I am very thankful for their help, it was very hard needing others to care for me. I had been independent all my life. Honestly, my quality of life was so poor. But after starting treatment, all my symptoms greatly improved. I am now fully independent again, I am eating well and sleeping well. Everything is good now! My follow-up has been smooth sailing. Well, other than some mild bloating.”

**Informed consent:** the patient was informed about the case report, why her case was unique, and how her case would be an invaluable addition to the limited medical information available on her condition. She gave informed consent to have this case report written up and published.

## Discussion

Systemic sclerosis (SSc) is an uncommon multisystem connective tissue disease that affects the gastrointestinal (GI) tract in nearly 90% of patients. Half of these patients are symptomatic [1]. Genetics, cytomegalovirus infection, environmental factors such as silica or industrial fumes, and some cancer chemotherapy agents appear to play an aetiological role [2,3]. The disease is uncommon in Africa, although cases may be under-reported due to inaccessibility to healthcare [4].

A retrospective study with 50 Kenyan participants with systemic sclerosis done by Ilovi and Oyoo found that the mean age at diagnosis was 41.7 years with a male to female ratio of 1: 4 [3]. They also found that oesophageal disease (54%) was the fourth most common presentation after skin manifestation (100%), Raynaud´s phenomenon (64%) and pulmonary disease (56%) [3]. Our patient was a 74-year-old female who presented with 2 main symptoms, namely persistent GORD and chronic diarrhoea, as well as other gastrointestinal symptoms such as dysphagia, gastroparesis, chronic intestinal pseudo-obstruction (CIPO) and weight loss. The typical SSc signs of generalised skin hyperpigmentation and thickening, sclerodactyly, Raynaud´s phenomenon and usual interstitial pneumonitis were also present [1]. Our patient had a reactive ANA, which Ilovi and Oyoo found to be positive in 67% of the 21 Kenyan patients tested [3]. It is sensitive for diagnosis in approximately 95-100% of patients worldwide [5].

Globally, the oesophagus is the most common site of gastrointestinal manifestation involving 50-80% of patients [6]. Atrophy of the smooth muscle in the lower oesophageal sphincter and distal oesophagus as well as neuronal abnormalities result in hypomotility. This causes prolonged exposure to gastric acid with resultant GORD, often with complications of oesophagitis, ulcers, fibrotic strictures and Barrett´s metaplasia [7]. Upper endoscopy is indicated for evaluation of refractory heartburn, dysphagia, odynophagia and screening for Barrett´s oesophagus. High-resolution manometry can be used to assess oesophageal motility [7,8]. Proton pump inhibitors (PPI) and prokinetics are the mainstay management for GORD, and patients should also be advised on lifestyle changes for GORD [7,9]. Aspiration pneumonia can develop due to oesophageal dysmotility and may contribute to interstitial lung disease. It can be prevented by early and aggressive treatment of GORD [8,10]. Gastroparesis may also cause acid reflux due to impaired gastric emptying. Dietary modifications and prokinetic agents are the common therapies used for gastroparesis, and an upper endoscopy should be done to rule out alternative diagnoses [7].

The small bowel is the second most common site of gastrointestinal involvement with a 40-88% global prevalence [7,9]. The pathogenesis of dysmotility is similar to that in the oesophagus with resultant impaired peristalsis [7,9]. Up to one-third of patients are at risk of SIBO due to the disease or achlorhydria from PPI use, and may cause loose motions, steatorrhea, weight loss and malnutrition [7]. These were present in our patient. Diarrhoea may also be due to pancreatic exocrine insufficiency or overflow from constipation which may cause faecal incontinence [7]. Diagnosis of SIBO is made by a positive breath test or jejunal aspirate cultures, whereas exocrine pancreatic insufficiency is diagnosed using tests such as faecal fat and elastase as well as pancreatic imaging [7]. If diarrhoea is due to SIBO, antibiotic cycling is the mainstay of treatment. Pancreatic insufficiency is managed by oral enzyme replacement, whereas constipation is treated with stool softeners and occasional stimulant laxatives [7,9]. Colonoscopy is indicated for screening, refractory constipation or alarm features such as anaemia or rectal bleeding, while imaging is used to diagnose pseudo-obstruction [7].

## Conclusion

The gastrointestinal tract is often involved in systemic sclerosis, which may result in some patients presenting initially to gastroenterologists. Our patient had clinical features resulting from progressive dysmotility, and recommended treatment aims for symptomatic relief, prevention of complications and maintenance of adequate nutrition and electrolytes. Gastrointestinal involvement is associated with substantial morbidity and mortality, and the presence of malabsorption and oesophageal dysfunction is associated with an unfavourable prognosis. Clinicians are therefore encouraged to have a high level of suspicion in their differentials of persistent gastrointestinal dysmotility.
